# Functional connectivity in task-negative network of the Deaf: effects of sign language experience

**DOI:** 10.7717/peerj.446

**Published:** 2014-06-24

**Authors:** Evie Malaia, Thomas M. Talavage, Ronnie B. Wilbur

**Affiliations:** 1 Center for Mind, Brain, and Education, University of Texas at Arlington, TX, USA; 2 Weldon School of Biomedical Engineering, Purdue University, IN, USA; 3 School of Electrical and Computer Engineering, Purdue University, IN, USA; 4 Speech, Language, and Hearing Sciences, and Linguistics Program, Purdue University, IN, USA

**Keywords:** Functional connectivity, Task-negative network, Deaf, Sign language, American sign language, PCC, Inferior parietal cortex, Default network, Medial temporal gyrus

## Abstract

Prior studies investigating cortical processing in Deaf signers suggest that life-long experience with sign language and/or auditory deprivation may alter the brain’s anatomical structure and the function of brain regions typically recruited for auditory processing (Emmorey et al., 2010; Pénicaud et al., 2013 inter alia). We report the first investigation of the task-negative network in Deaf signers and its functional connectivity—the temporal correlations among spatially remote neurophysiological events. We show that Deaf signers manifest increased functional connectivity between posterior cingulate/precuneus and left medial temporal gyrus (MTG), but also inferior parietal lobe and medial temporal gyrus in the right hemisphere- areas that have been found to show functional recruitment specifically during sign language processing. These findings suggest that the organization of the brain at the level of inter-network connectivity is likely affected by experience with processing visual language, although sensory deprivation could be another source of the difference. We hypothesize that connectivity alterations in the task negative network reflect predictive/automatized processing of the visual signal.

## Introduction

Neurobiology of sign languages—natural languages that convey information in visual modality—is a testing ground for theories of language processing. Given that the brain, using the Task Negative network (TNN) (Fox et al., 2005), is constantly in a state of predictive monitoring for useful input, including language, it is important to address the question of how this monitoring is affected by experience with a visually-based sign language. Although there has been some work comparing the processing of meaningful visual stimuli, from gesture to pantomime, in both Deaf ^1^1Deaf with capital D means the participants were non-hearing signers, as well as culturally part of the Deaf community. and hearing participants (Nakamura et al., 2004; Xu et al., 2009; Emmorey et al., 2010, inter alia), and associated structural plasticity of the signing brain (Emmorey et al., 2003; Shibata, 2007; Li et al., 2012; Pénicaud et al., 2013, inter alia), no work has yet focused on the potential long-term changes to anticipatory-predictive activation in the TNN (Buckner, Andrews & Schacter, 2008; Buckner, 2012) in Deaf signers as related to visual language experience. The present study investigates the functional connectivity among TNN regions in Deaf signers and hearing non-signers to assess network-level adaptations to sign language processing.

The task-negative network is a set of brain regions that are relatively more active during wakeful rest, than in the presence of external task or stimuli.^2^2Task-negative network is also referred to as default state, or default mode network (DMN). We are using the term task-negative to emphasize the absence of task in the research paradigm; however, in the literature, the two terms are used interchangeably. Recent discussions of the task-negative activations, as well as default mode network activity in the human brain (cf. Raichle, 2011; Besle et al., 2011, for review), suggested that such activations serve an experience-related function of predictive attention between tasks or in the absence of a specific task, rather than simply reflecting the anatomical connectivity. Two studies (Lewis et al., 2009; Sala-Llonch et al., 2012) have demonstrated that learning (in perceptual or memory tasks, respectively) alters baseline brain activation, entraining spontaneous de-coupling activity in the regions related to the task. Both studies indicated that TNN activity correlates with cognitive and behavioral performance and changes with learning. Lewis et al. (2009) suggested that TNN acts “as a form of “system memory” that recapitulates the history of experience-driven coactivation on cortical circuitries”. How would life-long experience of using sign language for communication be reflected in this network?

We know that human ability to monitor the environment for meaningful signals can be affected by the native language modality (visual vs. auditory), changing the roles of, and the connectivity among, the nodes of TNN. Prior studies of Deaf signers showed increase in right-lateralized processing (cf. Newman et al., 2001) compared to hearing non-signers; however, those studies typically are confounded by the impossibility of using the same stimuli for both groups. However, if sign language processing requires more engagement of right hemisphere as compared to that of spoken language, then lifetime experience with ASL will alter functional connectivity of TNN in signers to indicate higher connectivity either among the nodes within the right hemisphere, or between right and left hemisphere nodes, as compared to non-signers.

The present study investigated this hypothesis by exploring the functional connectivity among the regions of interest (ROIs) identified within the task-negative network (TNN) in Deaf signers. To this end, we carried out functional connectivity analysis of TNN hubs in Deaf signers and hearing non-signers to explore changes in functional connectivity related to sign language experience.

## Methods

### Participants

Two participant populations included Deaf signers and hearing non-signers who were part of a larger study that also involved fMRI (Malaia et al., 2012). Seventeen healthy Deaf adults who were native/near-native ASL signers (10 male, 7 female; 18–58 years old, mean age 35.6, SD = 14.2) and twelve hearing non-signers (7 male, 5 female, 19–36 years old, mean age 24.1, SD = 4.5) participated for monetary compensation after giving written informed consent in accord with the Purdue University Institutional Review Board approval #0506002702. All of the included participants were right-handed; five Deaf and seven hearing participants were right-eye dominant. None of the participants had any history of head injury or other neurological problems, and all had normal or corrected-to-normal vision. All deaf participants had completed at least a high school education; eight had at least some college or beyond. Hearing participants had all completed high school and at least some college or beyond. IQ level information was not collected; standard procedures for assessing intelligence in deaf populations use non-verbal protocols, as verbal protocols are considered to be language assessments rather than pure intelligence, given that language deficiencies are the major consequence of early hearing loss. No standardized norms are known to exist for adults (as opposed to children). More critically, Deaf participants in this study were screened for (1) early or native learning of ASL, (2) education level, and (3) type of educational setting(s). While age of language acquisition is known to affect studies of various types, there is no evidence that education level or type of education setting affects any of the relevant tasks independent of age of language acquisition. They are, however, potentially critical to degree of language fluency, which was important for the ASL task and hence to inclusion in the study.

### Scanning protocol

**Figure 1:**
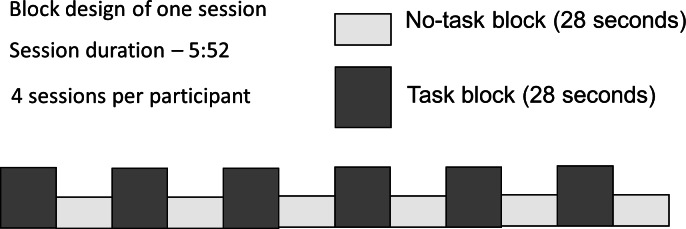
Block design with alternating Task and No Task conditions.

Participants were presented with dynamic video clip stimuli in a block paradigm (Fig. 1). During half of the 28-second blocks the participants were required to carry out an active task, which consisted of viewing video clips of ASL verb signs, and answering a question about them (Task), while the other half required only passive viewing (No Task). Participants responded to each stimulus with a button-press; the task was introduced to ensure behavioral compliance (that participants were awake and paying attention); the questions did not relate to those properties of the stimuli that were under investigation, thus there is no behavioral ‘result’ to report. Each participant took part in 4 sessions, lasting 5 min 52 s each.

The stimuli were displayed to participants via Nordic NeuroLab Visual System goggles (field of view: 30° horizontal, 23° vertical). During the Task condition, participants responded to the stimuli by pressing buttons on an MRI-compatible response box (Current Designs LLC HH-2x4-C) with their left hand, using their index finger.^3^3Full details of the task are provided in (Malaia et al., 2012). The duration of No-task blocks was sufficient to identify TNN activation, since the network has been shown to engage rapidly in the absence of specific task (van den Heuvel et al., 2008).^4^4See also microstate analysis of concurrent EEG-fMRI recordings van de Ville et al. 2010, suggesting that the dynamics of brain activation is fractal (or scale-free) in the time domain. Data collected from five of the Deaf participants were discarded: two due to equipment malfunction, one due to left-handedness (Oldfield, 1971), one participant only provided data for 2 of the 4 runs, and one did not provide behavioral responses; data from one hearing participant was also discarded due to recording issues; analyzed data set included 12 Deaf signers and 11 hearing non-signers.

All imaging data were collected on a 3 T GE Signa HDx (Purdue University MRI Facility, West Lafayette, Indiana), with 3D FSPGR high-resolution anatomical images (FOV = 24 cm, 186 sagittal slices, 1 mm × 1 mm in-plane resolution, slice thickness = 1 mm) acquired prior to functional scans. Functional scans were collected using a gradient echo-planar imaging sequence (TE = 22 ms, TR = 2 s, FOV = 24 cm, FA = 70°, 26 contiguous slices with 4 mm thickness, and 3.8 mm × 3.8 mm in-plane resolution; 176 time points). Four runs of this sequence were used to collect functional data for each participant.

### Data processing

Preliminary fixed effects analysis of functional imaging data was carried out using SPM5 software (http://www.fil.ion.ucl.ac.uk/spm). First, the initial 6 acquired volumes were removed to account for scanner stabilization, and each subject’s data were motion corrected to the 7th acquired volume; volumes associated with excessive head movement (more than 1 mm displacement between successive acquisitions) were eliminated. Data were then normalized to the standard Montreal Neurological Institute (MNI) space using the *T*_2_-weighted template provided by the SPM5 software and resliced to 2 × 2 × 2 mm^3^. Image registration was manually tested after the normalization process to verify the validity of this process. Each subject’s *T*_1_-weighted whole brain anatomical image was coregistered to the *T*_1_ weighted template provided by SPM5, and segmented to extract the gray matter maps. These maps were then optimally thresholded using the Masking toolbox of SPM5 to produce binary masks to be used as explicit masks in subsequent analyses. The last pre-processing step consisted of smoothing the functional data with an isotropic Gaussian filter (FWHM = 8 mm) to compensate for anatomical variability between subjects, and to match the statistical requirements of the general linear model.

Individual participant analyses were first performed in all subjects in order to identify the areas of the brain differentially activated during Task and No Task periods. For each subject, *t*-statistic maps were computed using a general linear model in SPM5, incorporating the six motion parameters as additional regressors. Specifically, brain activation for the No Task condition was contrasted against activation for the Task condition. The individual contrasts for Deaf and hearing group participants were then used as the input to between-participant analysis in SPM5 to obtain group results. The anatomical regions, maximum *t* values, MNI coordinates, and cluster sizes of the significant activation regions (*p* < 0.05, corrected for false discovery rate; number of voxels ≥10) for No Task vs. Task as revealed by random-effects analysis were identified.

### Data analysis

Functional connectivity analysis was performed on pre-processed fMRI data using partial correlation based on ICA after global signal regression.^5^5See Newman et al., 2013 for full details on the methodology. Seed regions of interest (ROIs) - spheres with 5 mm radius—were centered in peak task-independent deactivation coordinates from the Meta-Analysis from Laird et al. (2009), as two midline (posterior cingulate cortex, PCC [−4 −52 22], anterior cingulate cortex, ACC [2 32 −8]) and two lateral clusters in each hemisphere (right inferior parietal lobe (rIPL) [52 −28 24], left inferior parietal lobe (lIPL) [−56 −36 28]; right middle temporal gyrus (rMTG) [46 −66 16], left middle temporal gyrus (lMTG) [−42 −66 18]).^6^6The full nomenclature of default network nodes in Laird et al. (2009) includes, in addition to the listed nodes, precuneus, Medial Prefrontal Cortex, and left Middle Frontal Gyrus. The present study focused on the ROIs that were reliably identified in both populations as more active in No Task condition (see Table 1). For each participant the voxel timecourse in the ROIs was regressed against the time series for the motion correction parameters and global signal of the whole brain. Partial correlation analysis (regressing out time series from the other ROIs) was performed on each pair of regions using the first component of independent component analysis (ICA) in the signals from individual ROIs. Z-scores were then computed from the Pearson product-moment correlation coefficients for each ROI pair for each participant using Fisher r-to-Z transformation. Pairwise regional connectivity among TNN hubs in Deaf and hearing participants was then compared using independent-samples *t*-test in SPSS 15.

**Table 1:** Cortical areas activated in No Task condition in Deaf and hearing participants.

Anatomical region	Cluster size	Side	BA	Peak *t* value	Peak voxel coordinates	Cluster *p*-values, uncorrected	Cluster *p*-values, FDR-corrected
***Deaf***							
Anterior cingulate	514		24/32	4.94	−8 40 8	0.000	0.003
Insula	32	L	13	4.15	−40 −10 10	0.137	0.004
Occipital lobe	22	L	19	4.10	−28 −92 26	0.213	0.005
Parietal cortex/Posterior cingulate/precuneus	1837	R	5/7/31	4.93	4 −44 46	0.000	0.032
MFG	22	L	8	3.39	−26 22 46	0.213	0.019
SFG	108	R	8	3.94	16 32 48	0.012	0.006
Parieto-occipital junction	11	R	7	3.58	24 −78 48	0.378	0.013
***Hearing***							
Inferior temporal gyrus	32	L	20/21	3.67	−54 −8 −26	0.116	0.030
Parahippocampal/Fusiform gyri	326	L		4.82	−24 −38 -18	0.000	0.012
MTG/ITG/STG	130	R	20/21	4.06	56 −6 −8	0.004	0.020
Parahippocampal/Fusiform gyri	130	R		3.81	28 −44 −10	0.004	0.027
Anterior cingulate	668		10/24/32	4.57	−6 34 0	0.000	0.013
Lingual gyrus	12	L		3.28	−10 −80 −6	0.326	0.032
Lingual gyrus	15	R		3.35	14 −82 −2	0.272	0.031
STG	14	R	22	3.25	60 −16 2	0.289	0.033
Posterior cingulate/Parietal lobe	1244	L	7/19/31	4.12	12 −54 20	0.000	0.018
MFG	173	R	9	3.67	8 52 20	0.001	0.030
Angular gyrus	178	R	39	5.08	50 −74 32	0.001	0.011
Occipital lobe	22	L	19	3.38	−18 −92 28	0.187	0.031
Angular gyrus	15	L	39	3.35	−48 −68 30	0.272	0.031
Occipito-parietal junction	38	L	19/39	3.58	−44 −78 34	0.089	0.031
MFG	102	R	8	4.08	26 24 44	0.009	0.020
SFG	158	L	8	3.85	−24 32 54	0.002	0.026

**Notes.**

For each cluster, the peak location is given in MNI coordinates, accompanied by location in terms of Brodmann’s area and sulcal/gyral locus. *T* values represent the peak voxel activation within each cluster.

## Results

### Task-negative network in Deaf signers and hearing non-signers

**Figure 2:**
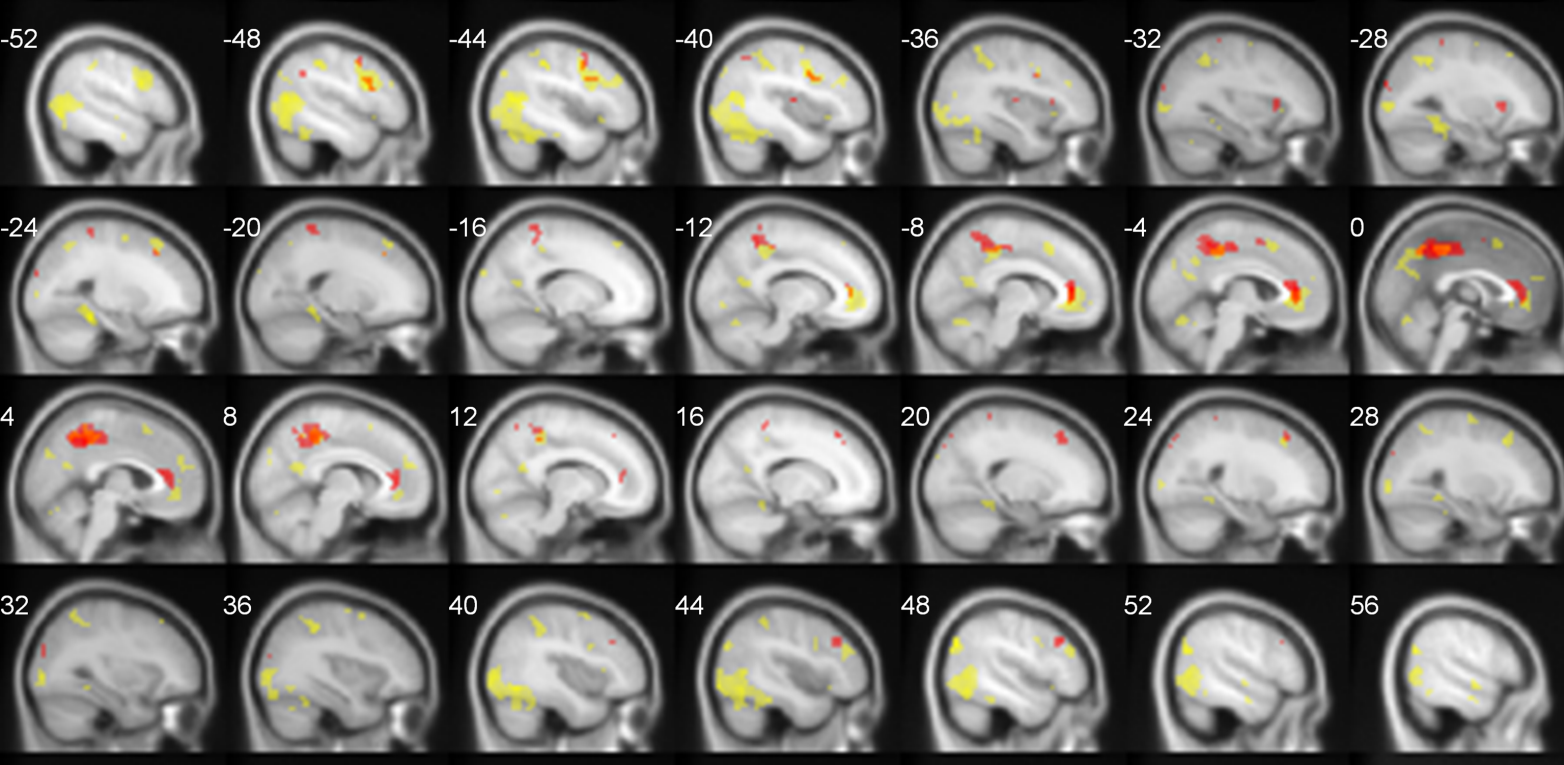
TNN activations (No Task > Task) in hearing (yellow) and Deaf (red) participants. FDR-corrected, *p* < 0.05.

The summary of neural activations for TNN in Deaf signers and hearing non-signers is presented in Table 1. Overall, TNN activations in both populations conformed to the typical expectations of TNN, or default mode network, incorporating regions along the anterior and posterior midline (anterior cingulate/ACC, posterior cingulate/PCC), and inferior parietal (IPL^7^7Inferior parietal lobe (IPL) includes the portion of the cortex that lies below the horizontal segment of the intraparietal sulcus, and behind the lower part of the postcentral sulcus. In the table for Deaf participants, IPL activation is reported in combined clusters with adjacent activation in the occipital cortex.) and dorsomedial prefrontal (dMPFC) cortices in left and right hemispheres. However, Deaf participants did not exhibit activation of Lateral Temporal cortex in TNN, while hearing ones did (Fig. 2).

### Functional connectivity analysis

Functional connectivity calculated after global signal regression using ICA was stronger in Deaf signers as compared to hearing non-signers between the following regions: PCC and left MTG (*t* = 3.829; *p* < .001); right IPL and right MTG (*t* = 12.932, *p* < .001). The connectivity between the following regions was higher in non-signers than signers: PCC and right MTG (*t* = 8.934, *p* < .01), and right IPL and left MTG (*t* = 3.707, *p* < .002) (see Table 2). No other differences in functional connectivity between ROIs were observed.

**Table 2:** Pairwise functional connectivity between regions that was significantly stronger in Deaf signers or hearing non-signers in comparison between the two groups. No other differences were observed in functional connectivity between network components.

Deaf signers	*t*	*p* <	Hearing non-signers	*t*	*p* <
PCC - left MTG	3.829	.001	PCC - right MTG	8.934	.01
Right IPL- right MTG	12.932	.001	Right IPL – left MTG	3.707	.002

## Discussion

Higher functional connectivity between rIPL and rMTG clusters in Deaf signers vs. hearing non-signers suggests that the parietal cortices in the Deaf might be used to process components of the visual linguistic signal, indicating experience-based difference in processing networks for dealing with systematic input.

### Functional connectivity across regions in the right hemisphere

In Deaf signers, two TNN nodes within the right hemisphere—rIPL and rMTG—showed higher functional connectivity than in hearing non-signers. Although increased right hemisphere activation has been an important issue in studying the neural basis of sign language processing (Hickok, Love-Geffen & Klima, 2002; Neville et al., 1998), our analysis further confirms the network-level relevance of right hemisphere activations as part of the anticipatory response in sign language users, as IPL and MTG were specifically identified here as portions of the TNN. The meta-analysis by Laird et al. (2009) notes increase in rIPL-rMTG connectivity as a part of somatosensory perception network. While our analysis does not directly explain why right hemisphere activation is specifically necessary for the processing of visual language, one possibility might have to do with the fractal complexity of sign language input across spatiotemporal scales (Malaia et al., 2012; Bosworth, Bartlett & Dobkins, 2006)—a feature in which sign language input in the visual domain is similar to musical input in the auditory domain, and which might be the reason for right-hemisphere neural recruitment for binding of perceptual fragments across temporal scales into a unified percept.

### Role of PCC in TNN

In Deaf signers, PCC showed stronger correlation with left MTG than in hearing non-signers. Prior analyses of PCC’s role in language processing (Malaia et al., 2012; Malaia, 2014; Newman et al., 2013) suggested that it is crucial for event schema retrieval, as its recruitment increases with processing strategies requiring unification of working memory contents. Default mode network investigation (Sala-Llonch et al., 2012), which observed increased functional connectivity of PCC related to behavioral improvement on an n-back WM task, suggested that PCC might act as a part of the orienting attentional network, primed as part of the default mode network activity to increase task-related capacity for integration of complex stimuli in subsequent tasks. Both explanations of PCC’s role concur that it is the increased role of PCC *during* the task (as observed in Malaia et al., 2012) which leads to its increased functional connectivity with task-relevant processing regions in TNN.

### Implications for theories of language processing

The contribution of the data on TNN activation and functional connectivity to the current literature on the dorsal/ventral pathway analysis in processing of linguistic and visual information (Bornkessel-Schlesewsky & Schlesewsky, 2013; Nakamura et al., 2004) is the indication that activation of the lateral temporal cortices is likely modality-specific, as observed in the present study. To date, the task-related function of the temporal lobe in Deaf signers has been found to be similar to that of hearing non-signers inasmuch as non-auditory processing is concerned: it includes modality-independent phonetic processing, verbal memory, and other language functions (Emmorey, Xu & Braun, 2011; Malaia & Wilbur, 2010). Additionally, functional connectivity analyses of TNN in hearing populations show that LTC activation has the weakest correlation with the other hubs in the default mode activation (Buckner, Andrews & Schacter, 2008; Andrews-Hanna et al., 2010), suggesting that it might not be central to TNN’s function.

Additionally, the observation that lifelong visual language experience leads to changes in TNN that include an increase in connectivity of right IPL and MTG cortices, and PCC and left MTG contributes to the laterality debate surrounding sign language processing (Neville et al., 1998; MacSweeney et al., 2002; Allen et al., 2008; Emmorey et al., 2010), suggesting that the increase in bilateral activation during sign language processing, as compared to spoken language, is not task-specific. Rather, repeated exposure to, and practice in comprehension of, sign language appear to lead to profound alterations in functional connectivity, as demonstrated by our data, as well as structural changes, such as increase in right hemisphere white matter volume (Allen et al., 2008).

One possible question that can be raised is whether the findings might be due not to sign language experience, but auditory deprivation instead. One recent study that evaluated participants with varying levels of auditory deprivation and sign language experience (Cardin et al., 2013) found that auditory deprivation effects are localized to occipital and superior temporal cortex. The study did not address the functional connectivity question directly, however, and thus might not have detected the changes reported here. Thus, we cannot discard the possibility that auditory deprivation contributed, directly or indirectly, to the observed pattern of functional connectivity in Deaf signers. At the same time, the observed results cannot be explained by changes in visual or auditory components of resting state network, as identified by probabilistic ICA (Damoiseaux et al., 2006), allowing for higher likelihood that they are, in fact, due to cognitive experience of using sign language. Also, a recent study (Olulade et al., 2014) showed that anatomical differences attributed to auditory deprivation vary depending on whether the deaf participants are native users of sign language or not, indicating the difficulties involved in deconvolving the effects of sensory and linguistic variables.

## Conclusion

Analysis of the task-negative network activity in Deaf signers demonstrates that visual language experience is associated with increased correlation in the activity of the precuneus/posterior cingulate and left MTG, as well as higher functional connectivity between right IPL and MTG - areas that have been found to show functional recruitment during visual language processing and event schema retrieval. These findings suggest that experience with processing visual language, and subsequent connectivity alterations in the default mode network aimed at predictive/automatized processing of the visual signal, affects organization of the brain at the level of inter-network connectivity. Future studies with hearing signers will be needed to determine with certainty whether the observed differences in functional connectivity between Deaf signers and hearing non-signers are due to sensory deprivation, or sign language experience.
